# The effects of common variants in *MDM2* and *GNRH2* genes on the risk and survival of osteosarcoma in Han populations from Northwest China

**DOI:** 10.1038/s41598-020-72995-4

**Published:** 2020-09-29

**Authors:** Weilou Feng, Zhi Wang, Dongxu Feng, Yangjun Zhu, Kun Zhang, Wei Huang

**Affiliations:** 1 Department of Orthopaedic Trauma, HongHui Hospital, Xi’an Jiaotong University, 555 Youyi East Road, Beilin District, Xi’an, 710053 Shaanxi China; 2 Department of Neonatology, Xi’an Children’s Hospital, Xi’an, Shaanxi China

**Keywords:** Risk factors, Genetic predisposition to disease

## Abstract

Accumulating evidence has shown that both MDM2 and GNRH2 might be related to Osteosarcoma (OS) susceptibility. The study aimed to evaluate the effects of common variants in *MDM2* and *GNRH2* genes on the risk and survival of osteosarcoma in Han populations from Northwest China. In the study, we recruited 2292 subjects including 596 OS patients and 1696 healthy controls and genotyped 16 selected tag SNPs (6 from *GNRH2* and 10 from *MDM2*). Genetic association analyses were performed at the genotypic and allelic levels. Survival curves were made for OS patients with different genotypes. Two SNPs, rs1690916 *(MDM2, P* = 0.0002) and rs3761243 (*GNRH2, P* = 0.0004), were identified to be significantly associated with OS risk. Moreover, SNP rs3761243 was strongly associated with pathological fracture (*P* = 2.61 × 10^–14^), metastasis (*P* < 2.2 × 10^–16^), and Enneking stage (*P* < 2.2 × 10^–16^) in the OS group. Furthermore, survival curves based on different genotypes of SNP rs3761243 were found to be significantly different (*P* = 0.0003), suggesting increased risk with more copies of C alleles. Our results provide supportive evidence for genetic associations of *MDM2* and *GNRH2* genes with susceptibility to OS, and for the positive correlation of SNP rs3761243 in *GNRH2* with the survival status of OS patients in Han populations from Northwest China.

## Introduction

Osteosarcoma (OS) is a common osteogenic tumor that occurs in children and adolescents under 18 years of age^1^. Because of the high malignancy and aggressiveness of OS, the prognosis of OS is poor^2^. Surgery and chemotherapy are currently the main treatments for OS, but the 5-year survival rate of OS is still less than 50%^3^. Hence, determining the pathogeny of OS and clarifying the molecular mechanism of OS are important problems to be solved. Previously obtained evidence has shown that the development of OS is complex and would be affected by many factors^4,5^. However, the fact that only a portion of individuals exposed to the same environment would develop OS has caught the attention of researchers, indicating that genetic factors are also risk factors for OS. Therefore, it is necessary to determine the susceptibility genes of OS through further research.

Previous research has shown that overexpression of *MDM2* is found in a number of human cancers^6^. A case–control study has identified the significant association of genetic variants with OS in some candidate genes from biologically plausible pathways involved in growth, hormone metabolism, and DNA repair^7^. This study provided significant evidence for the associations of rs1690916 in the *MDM2* gene and rs3761243 in the *GNRH2* gene with OS^7^. Additional evidence has also been reported suggesting that the single nucleotide polymorphism (SNP) rs16909916 located in the 3′ untranslated region of *MDM2* is associated with susceptibility to OS in Europeans^8,9^. Nevertheless, some negative results for the association between rs16909916 and OS have also been reported. A case–control study did not observe any association between rs16909916 and OS in Spanish and Slovenian populations^10^. In addition, a meta-analysis including six populations also identified no association signals^10^. A previous study also detected a significant association of rs3761243 in *GNRH2* with bone neoplasms^11^. Given that the molecular mechanisms of OS have not been determined, even though there is evidence of significant associations with OS in Europeans, the contributions of *MDM2* and *GNRH2* to the pathogenesis of OS have not been fully explained to date. Thus, to characterize the roles of *MDM2* and *GNRH2* genes in OS predisposition, it is important to evaluate the relationship between them and OS risk in different ethnic populations.

To date, the roles of *MDM2* and *GNRH2* in the risk of OS in Han populations from Northwest China have not been explored. Thus, we aimed to examine the associations of common variants in *MDM2* and *GNRH2* with the OS susceptibility and to provide new clues for identifying individuals at high risk for OS and developing preventive strategies.

## Materials and methods

### Study subjects

We recruited a total of 2292 study subjects, consisting of 596 OS patients and 1696 controls from Honghui Hospital of Xi’an Jiaotong University and Xi’an Children’s Hospital between January 2011 and April 2019. All OS patients were diagnosed based on standard histological and pathological evidence according to a consensus reached by at least two pathologists. The OS patients were followed after diagnosis, and death and month of survival of OS patients were recorded during the study period. Healthy controls included individuals who attend routine medical examination in the same hospitals mentioned above. To ensure the homogeneity of the genetic background, our study subjects were restricted to Xi'an natives with no immigration history within the last three generations. Informed consent forms were obtained from all participants. This study was performed in accordance with the ethical guidelines of the Helsinki Declaration of 1975 (revised in 2008) and was approved through the Ethics Committee of Honghui Hospital of Xi'an Jiaotong University (NO. 20190123).

### SNP selection and genotyping

We selected tag SNPs for genotyping according to the following three criteria: (1) SNPs with minor allele frequency (MAF) ≥ 0.05; (2) SNPs located within ± 3 kb gene regions of *MDM2* and *GNRH2*; and (3) tag SNPs with r^2^ ≥ 0.8. In total, 16 tag SNPs were finally obtained for subsequent genotyping (6 from *GNRH2* and 10 from *MDM2*). Basic information on these SNPs is summarized in Supplemental Table S1. We used commercial kits (Genomic DNA kit, Axygen Scientific, Inc., CA, USA) to extract genomic DNA from peripheral blood leukocytes. Then, a total of 16 SNPs were genotyped based on a high-throughput Sequenom MassARRAY platform. The genotype data of all SNPs in each subject were obtained. During the quality control phase, we randomly selected 5% of the samples for repeated genotyping^12^. The results are exactly the same as before, confirming the reliability of the quality of the genotyping data. During genotyping, the case/control status of each sample is unknown to all technicians^13^.

### Statistical analysis

Hardy–Weinberg equilibrium (HWE) tests were conducted in the control group. Linkage equilibrium (LD) plots of the selected SNPs were constructed using Haploview^14^. Genetic association analyses were performed at the genotypic and allelic levels. For genotypic analysis, Cochran-Armitage trend tests were performed. For allelic analysis, χ^2^ tests were conducted. In addition, related analyses were preformed to examine the correlation between associated SNPs and several clinical features, including tumor location, pathological fracture, metastasis status and Enneking stage, within the OS patient group. Plink was utilized for genetic association analysis^15^. Bonferroni correction was used in multiple tests. For the scenario of SNP association analysis, when the *P* value was less than 0.05/16 = 0.003125, it was considered to be significant. Survival analyses were performed for OS patients with different genotypes of significant SNPs. Survival curves were made for OS patients with different genotypes. The Kaplan–Meier estimator and the log-rank test were utilized to evaluate the differences in survival curves for different genotypes. Cox models were fitted to estimate the hazard ratio of specific genotypes. The R project with the "survival" package (v3.2-3) was utilized for survival analysis^16^. In order to examine the functional consequence of the associated SNPs, we investigated the potential expression quantitative trait loci (eQTL) signals of the significant SNPs using data obtained from the GTEx database^17^. The data of eQTL signals for relevant SNPs on *MDM2* and *GNRH2* from multiple human tissues were downloaded and analyzed. In addition, we examined the significant SNPs in the PolymiRTS Database to explore the role of the SNPs in miRNA binding^18^.

## Results

### Demographic and characteristic information of the study subjects

In the present study, we recruited 596 OS patients and 1696 control subjects (Table 1). No significant differences could be identified for gender (*P* = 1.00) and age (*P* = 0.4374) between cases and controls. A significant difference was identified for family history between cases and controls (*P* = 6.80 × 10^–11^). Among the 596 OS patients, 114 (19%) had tumors at their axial bones, and 482 (81%) had tumors in their long bones. A total of 106 OS patients (18%) had pathological fractures, and 490 patients (82%) did not. Seventy-eight patients (13%) had metastasis, and 518 patients (87%) did not. Twelve percent of the OS patients (72) were in stage I of the Enneking staging system, and 75% (446 OS patients) and 13% (78 OS patients) were in stage II and stage III, respectively.

**Table 1:** Demographic and characteristic information of the study subjects.

Variables	Cases (N = 596)	Controls (N = 1696)	Statistics	*P*-values
Age, mean + sd	21.3 ± 5.2	21.5 ± 5.3	*T* = − 0.78	0.44
**Gender, N (%)**				
Male	314 (53)	892 (53)		
Female	282 (47)	804 (47)	χ^2^ = 0.00	1.00
**Family history, N (%)**				
Yes	66 (11)	64 (4)		
No	530 (89)	1632 (96)	χ^2^ = 42.58	6.80 × 10^–11^
**Tumor location, N (%)**				
Axial	114 (19)			
Long	482 (81)			
**Pathological fracture, N (%)**				
Yes	106 (18)			
No	490 (82)			
**Metastasis, N (%)**				
Yes	78 (13)			
No	518 (87)			
**Enneking stages, N (%)**				
I	72 (12)			
II	446 (75)			
III	78 (13)			

### Genetic association signals between genetic polymorphisms and OS risk

HWE tests were performed in control group. These SNPs were all in HWE in controls (Supplemental Table S1). LD plots are shown in Supplemental Figures S2 and S2. Among the 16 genotyped SNPs, two SNPs, rs1690916 *(MDM2*) and rs3761243 (*GNRH2*), were determined to be strongly associated with the risk of OS (Table 2). Similar significant results were also obtained in genotypic analyses. For SNP rs1690916, its A allele was correlated with the decreased risk of OS (OR = 0.73 [0.61–0.86], χ^2^ = 13.99, *P* = 0.0002). On the other hand, the C allele of SNP rs3761243 was positively associated to an increased risk of OS (OR = 1.27[1.11–1.45], χ^2^ = 12.54, *P* = 0.0004). Clear dose-dependent effects could be observed in genotypic analyses for both SNPs. The effect size increased when more copies of targeted alleles were present (Table 2).

**Table 2:** Significant SNPs identified in single marker based association analyses.

	CHR	Gene	Genotypic Analyses						Allelic Analyses					
			Genotypes	Cases (%)	Controls (%)	OR [95%]	*T*	*P*	Alleles	Cases (%)	Controls (%)	OR [95%]	χ^2^	*P*
rs1690916	12	*MDM2*	AA	13 (2)	93 (5)	0.36 [0.20–0.64]								
			AG	185 (31)	590 (35)	0.80 [0.65–0.98]			A	211 (18)	776 (23)	0.73 [0.61–0.86]		
			GG	398 (67)	1013 (60)	Ref	14.00	0.0002	G	981 (82)	2616 (77)	ref	13.99	0.0002
rs3761243	20	*GNRH2*	CC	168 (28)	377 (22)	1.58 [1.22–2.06]								
			AC	286 (48)	814 (48)	1.25 [0.99–1.57]			C	622 (52)	1568 (46)	1.27 [1.11–1.45]		
			AA	142 (24)	505 (30)	Ref	12.07	0.0005	A	570 (48)	1824 (54)	ref	12.54	0.0004

CHR: chromosome.

### Association between genetic polymorphisms and clinical features in OS patients

For the two significant SNPs, we examined their associations with several clinical features within the OS patient group (Table 3). SNP rs1690916 was not significantly associated with any clinical features in the OS group. However, SNP rs3761243 was significantly associated with pathological fracture (χ^2^ = 62.56, *P* = 2.61 × 10^–14^), metastasis (*P* < 2.2 × 10^–16^), and Enneking stage (*P* < 2.2 × 10^–16^) in the OS group. These results suggested clues for further survival analysis.

**Table 3:** Association between significant SNPs and clinical variables in cases.

	rs1690916			χ^2a^	*P*	rs3761243			χ^2^	*P*
	AA	AG	GG			CC	AC	AA		
**Tumor location, N (%)**										
Axial	1	40	73			39	55	20		
Long	12	145	325	–	0.40	129	231	122	4.15	0.13
**Pathological fracture, N (%)**										
Yes	2	32	72			63	31	12		
No	11	153	326	–	0.97	105	255	130	62.56	2.61 × 10^–14^
**Metastasis, N (%)**										
Yes	2	20	56			76	1	1		
No	11	165	342	–	0.57	92	285	141	–	< 2.2 × 10^–16^
**Enneking stages, N (%)**										
I	4	28	40			2	2	68		
II	7	137	302			90	283	73		
III	2	20	56	–	0.06	76	1	1	–	< 2.2 × 10^–16^

^a^Fisher exact tests were performed for sparse data.

### Survival analysis

**Figure 1:**
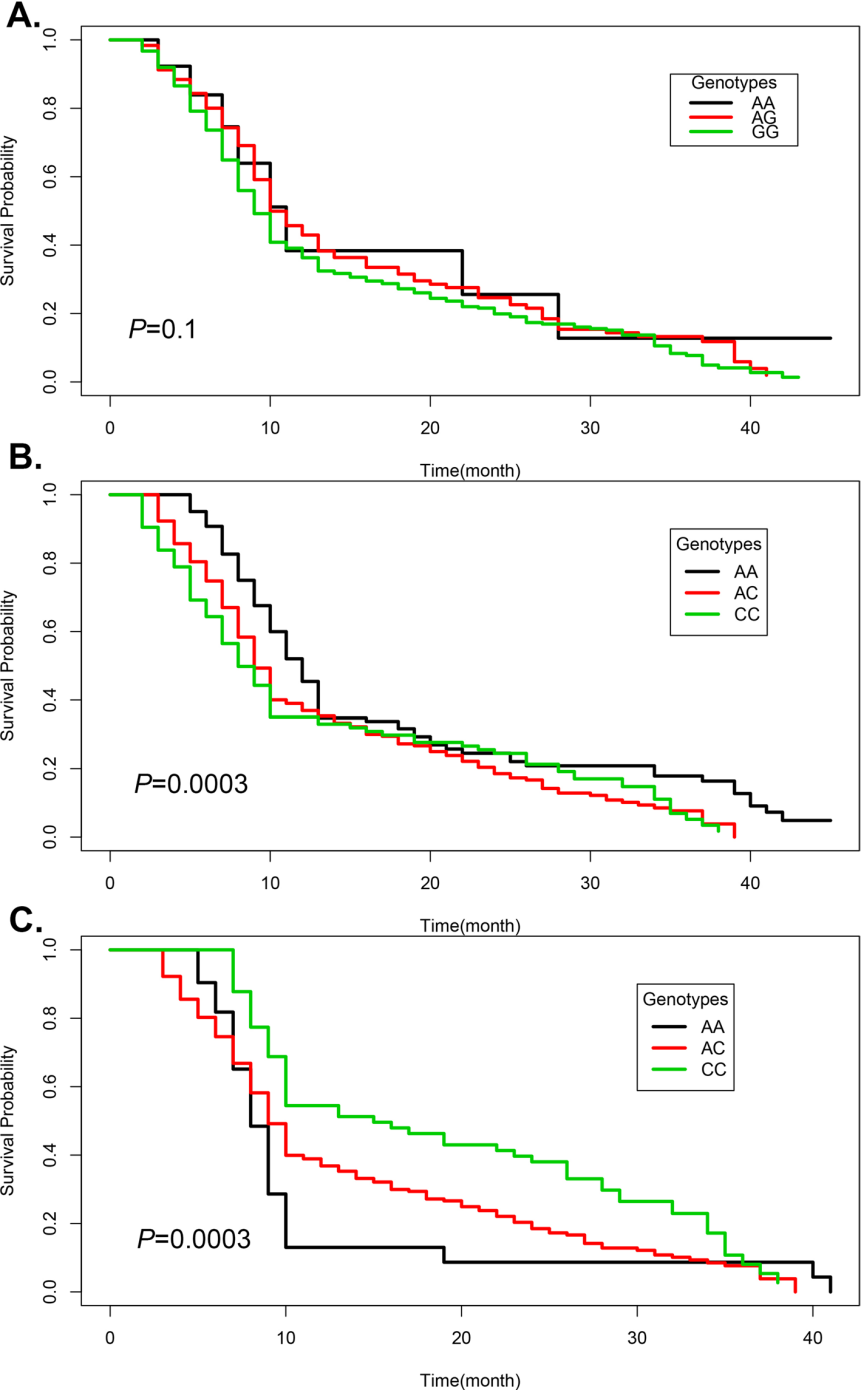
Survival curves for different groups of OS patients. (**A**) Survival curves categorized by genotypes of SNP rs1690916. (**B**) Survival curves categorized by genotypes of SNP rs3761243. (**C**) Survival curves categorized by genotypes of SNP rs3761243 in OS patients at Enneking stage II.

Survival curves are shown in Fig. 1. Survival curves based on different genotypes of SNP rs3761243 were determined to be significantly different (*P* = 0.0003, Fig. 1B). A similar difference was identified for survival curves made solely based on stage II OS patients (Fig. 1C). The Cox model for SNP rs3761243 was fitted for stage II OS patients with age, gender, pathological fracture, and metastasis status adjusted (Table 4). With the CC genotype serving as a reference, the hazard ratios with 95% confidence intervals for AC and AA genotypes were 0.89 [0.65–1.22] and 0.53 [0.36–0.78], respectively. Similar to the genotypic association analysis, a dose-dependent effect was also observed in the survival analysis. The hazard ratio increased when more copies of C alleles were present.

**Table 4:** Results of Cox model fitted for SNP rs3761243 using stage II osteosarcoma cases.

SNP	CHR	GENE	Genotypes	Hazard ratio [95%]	Z	*P*
rs3761243	20	*GNRH2*	CC (N = 90)	Ref	–	–
			AC (N = 286)	0.89 [0.65, 1.22]	− 0.72	0.4714
			AA (N = 70)	0.53 [0.36, 0.78]	− 3.23	0.0012

Fracture and metastasis status were adjusted in Cox model.

### Functional consequences of the significant SNPs

**Figure 2:**
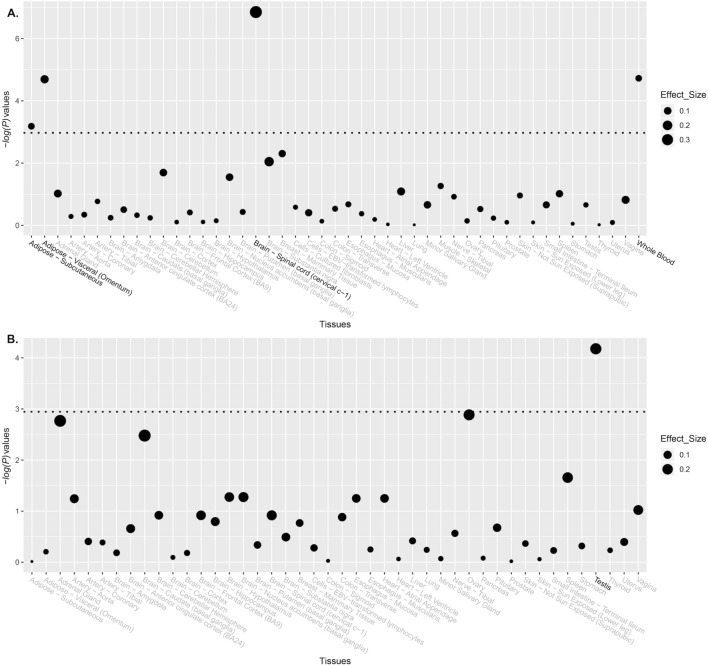
eQTL signals for both SNP rs1690916 and rs3761243 on *MDM2* and *GNRH2*, respectively. (**A**) eQTL signals for both SNP rs1690916 on *MDM2*. (**B**). eQTL signals for both SNP rs3761243 on *GNRH2.*

Based on the results obtained from the PolymiRTS Database, the A allele of SNP rs1690916 could create a new binding site for miR-1303 on gene *MDM2*. This miRNA is encoded by gene *MIR1303* located on 5q33.2. We explored the eQTL signals for both SNP rs1690916 and rs3761243 on *MDM2* and *GNRH2*, respectively (Fig. 2). Significant eQTL signals were identified for both SNPs in specific human tissues. SNP rs1690916 was strongly correlated with the gene expression of *MDM2* in the spinal cord, whole blood and adipose tissue. SNP rs3761243 was determined to be significantly associated with the expression level of *GNRH2* gene in the human testis.

## Discussion

With the widespread application of sequencing technology, numerous susceptible genes were identified to be contributed to the risk of complex diseases, such as schizophrenia^19–21^. The gene *MDM2,* located at 12q14.3–15, is an important negative regulator of TP53, inhibiting the transcriptional activity of TP53 and enhancing proteolytic P53 degradation^22,23^. The importance of the p53 pathway in maintaining normal cell growth is supported by the fact that it is directly inactivated by mutations in the *TP53* gene in approximately 50% of cancers^24^. Hence, *MDM2* plays a key role in the development of malignant tumors. The *GNRH2* (gonadotropin-releasing hormone 2) gene located at 20p13 participates in promoting gonadotropin synthesis and steroid hormone production^25^, and *GNRH2* is also emerging as an important player in the biology of cancers, such as prostate cancer and ovarian cancer^26,27^.

Our study is the first one to explore the relationship between genetic polymorphisms of *MDM2* and *GNRH2* and susceptibility of OS based on populations with Chinese ancestry. In the present study, we obtained significant evidence to connect the two loci and OS. The direction of the genetic effect identified in our study was the same, and the effect size was also highly similar, compared to the study conducted by Mirabello et al.^7^. In this sense, our study could be considered a successful replication of this previous report in a different population. Moreover, it is difficult to draw solid conclusions only from SNP results^28–30^. Thus, considering the genetic heterogeneity of OS and differences in LD structure among different populations, this replication was meaningful. In addition, in our study, we moved one step further to show the dose-dependent pattern of the genotypic effects for OS susceptibility.

In bioinformatics analysis, we have identified that the A allele of SNP rs1690916 could create a new binding site for miR-1303 on gene *MDM2*. A previous study has showed that miR-1303 targets claudin-18 gene to modulate proliferation and invasion of gastric cancer cells^31^. In addition, according to our eQTL analysis, both significant SNPs rs1690916 and rs3761243 were significantly associated (at least in some specific types of human tissues) with gene expression of *MDM2* and *GNRH2*, respectively. This finding indicated that both SNPs might have important functional consequences on the downstream translation from DNA variants to biological functions. Nevertheless, caution is required in interpreting the eQTL signals. In our study, our eQTL analyses were conducted solely based on data from a public database. As far as we know, there might be no OS patients included and tested in GTEx. However, the gene expression pattern and eQTL features might be significantly different in OS patients compared to control patients. In this sense, our eQTL signals identified based on GTEx data may only provide us with some clues but may not be used as solid evidence. In the future, an eQTL study based on OS patients is still needed to comprehensively examine the potential effect of SNPs rs1690916 and rs3761243 on the gene expression of *MDM2* and *GNRH2*.

We identified a strong correlation between the genotypes of SNP rs3761243 and the Enneking stages of OS in our samples. This observation is highly unusual because it seems that most OS patients in the stage I group had the AA genotype, while most patients in the stage III group had the CC genotype. Since the recruitment process of OS patients in our study was independent of the genotypes of SNP rs3761243 and the Enneking stages of the patients, a potential explanation for this observation is that SNP rs3761243 was highly correlated with Enneking stages in OS patients. However, more studies are still needed to replicate our results in other populations.

To date, no previous studies have focused on the survival of OS patients with different genotypes of SNP rs1690916 and rs3761243. Our survival analysis showed that the genotypes of SNP rs3761243 were significantly related to the survival of OS patients. In a further analysis focusing only on Enneking stage II OS patients, the AA genotype group had the poorest survival outcome. The HR estimated from the Cox model indicated that the C allele of SNP rs3761243 was positively correlated with a decreased hazard of OS death. This observation is highly interesting because the C allele at the SNP was correlated with an elevated risk of OS in genetic association analysis (OR = 1.27, Table 2), and it was also associated with elevated Enneking stages in OS patients (Table 3). Since SNP rs3761243 was not polymorphic in our Enneking stage I and Enneking stage III patients, we could not comprehensively analyze the effect of genotypes on the survival of OS patients in the present study. The results obtained from stage II patients might be inaccurate and biased. In the future, a well-balanced study design is needed to analyze the genotypic effects on the survival of OS patients.

Our study has several limitations. First, we only selected and genotyped 16 SNPs for the two candidate loci, and the information coverage might not be enough to thoroughly examine the genetic susceptibility of the two loci contributing to the risk of OS. In addition, only common polymorphisms were included in this study. However, recent genetic studies suggest that low frequency and rare genetic variations may play a more critical role in the pathogenesis of complex diseases, especially for cancers^32,33^. Therefore, sequence-based studies would be desired to completely examine the genetic architecture of both *MDM2* and *GNRH2*. Another potential limitation is that population stratification might be a confounded significant hit in the present study. Because of limited genotype data in the study, we hardly apply some standard statistical procedures that are commonly used in genome-wide association studies. Nevertheless, in the sample recruitment process, we restricted the genetic background of our study subjects by including only Xi’an natives with no immigration history in the last three generations. We think that this recruitment strategy at least partly addresses the potential confounding effect. In addition, the treatment protocols for patients with OS were not exactly the same in the study. These differences in treatment might affect the time of survival of these patients. Therefore, our results should be considered to be preliminary and validated in the future research.

The results of our study present significant evidence for a genetic association between OS susceptibility and *MDM2* and *GNRH2* genes in the sample of Han populations from Northwest China*.* More than genetic association with OS status, the genotypes of SNP rs3761243 in *GNRH2* were also significantly associated with the survival status of OS patients at Enneking stage II. Our findings help to elucidate the screening and diagnosis of OS and the prognosis of OS after regular treatment.

## Supplementary information

Supplementary file 1
