# Supplementary material for: The effects of common variants in MDM2 and GNRH2 genes on the risk and survival of osteosarcoma in Han populations from Northwest China
Source: Sci Rep. 2020 Sep 29;10:15939. doi: 10.1038/s41598-020-72995-4 (PMC7524757; doi:10.1038/s41598-020-72995-4)
Supplement: Supplementary file 1 [file 41598_2020_72995_MOESM1_ESM.docx]

***Title*:** The effects of common variants in *MDM2* and *GNRH2* genes on the risk and survival of osteosarcoma in Han populations from Northwest China

***Running title*:** *MDM2* and *GNRH2* genes and osteosarcoma

***Author names and affiliations***: Weilou Feng ^1^, Zhi Wang ^2^, Dongxu Feng ^1^, Yangjun Zhu ^1^, Kun Zhang ^1^ and Wei Huang ^1^

^1^ Department of Orthopaedic Trauma, HongHui Hospital, Xi'an Jiaotong University, Xi’an, Shaanxi, China;

^2^ Department of Neonatology, Xi’an Children’s Hospital, Xi’an, Shaanxi, China.

***Correspondence***:

Wei Huang, Department of Orthopaedic Trauma, HongHui Hospital, Xi'an Jiaotong University, 555 Youyi East Road, Beilin District, Xi’an, Shaanxi, China, 710053.

Tel: 86-29-88418009; Fax: 86-29-88418009, E-mail: [weihuangxa@163.com](mailto:weihuangxa@163.com)

Supplemental Table S1. Basic information of the SNPs selected for genotyping.

| CHR | POS | SNP | FUNC | GENE | A1 | A2 | MAF | HWE |
| --- | --- | --- | --- | --- | --- | --- | --- | --- |
| 12 | 68808800 | rs2279744 | near-gene-5 | *MDM2* | T | G | 0.44 | 0.73 |
| 12 | 68815509 | rs11177383 | intron | *MDM2* | C | A | 0.12 | 0.74 |
| 12 | 68822574 | rs78064292 | intron | *MDM2* | A | G | 0.12 | 0.43 |
| 12 | 68824258 | rs2291857 | intron | *MDM2* | T | G | 0.34 | 0.55 |
| 12 | 68824920 | rs3730572 | intron | *MDM2* | C | G | 0.08 | 0.49 |
| 12 | 68828045 | rs3730597 | intron | *MDM2* | G | C | 0.22 | 0.52 |
| 12 | 68829986 | rs1695143 | intron | *MDM2* | G | C | 0.21 | 0.46 |
| 12 | 68833454 | rs4913469 | intron | *MDM2* | G | T | 0.15 | 0.78 |
| 12 | 68841626 | rs1690916 | untranslated-3 | *MDM2* | A | G | 0.22 | 0.58 |
| 12 | 68846022 | rs139487710 | near-gene-3 | *MDM2* | T | C | 0.08 | 0.49 |
| 20 | 3040958 | rs6138989 | intron | *GNRH2* | A | C | 0.35 | 0.63 |
| 20 | 3041494 | rs60692390 | intron | *GNRH2* | G | T | 0.34 | 0.48 |
| 20 | 3042376 | rs3761243 | intron | *GNRH2* | C | A | 0.48 | 0.16 |
| 20 | 3043726 | rs6138992 | intron | *GNRH2* | C | T | 0.12 | 0.81 |
| 20 | 3045423 | rs676749 | intron | *GNRH2* | T | A | 0.06 | 0.19 |
| 20 | 3048065 | rs1100 | untranslated-3 | *GNRH2* | A | G | 0.31 | 0.53 |

CHR: chromosome; POS: position; FUNC: function; A1: minor allele; A2: major allele; MAF: minor allele frequency; HWE: *P* values for Hardy-Weinberg equilibrium tests.

Supplemental Table S2. Full results of the single marker based association analyses for the genotyped SNPs.

| CHR | SNP | A1 | A2 | TEST | AFF | UNAFF | CHISQ | DF | *P* |
| --- | --- | --- | --- | --- | --- | --- | --- | --- | --- |
| 12 | rs11177383 | C | A | ALLELIC | 151/1041 | 421/2971 | 0.05302 | 1 | 0.8179 |
| 12 | rs11177383 | C | A | TREND | 151/1041 | 421/2971 | 0.05338 | 1 | 0.8173 |
| 12 | rs11177383 | C | A | DOM | 141/455 | 397/1299 | 0.01531 | 1 | 0.9015 |
| 12 | rs11177383 | C | A | REC | 10/586 | 24/1672 | 0.2084 | 1 | 0.6481 |
| 12 | rs139487710 | T | C | ALLELIC | 97/1095 | 261/3131 | 0.2404 | 1 | 0.6239 |
| 12 | rs139487710 | T | C | TREND | 97/1095 | 261/3131 | 0.2361 | 1 | 0.627 |
| 12 | rs139487710 | T | C | DOM | 92/504 | 249/1447 | 0.1983 | 1 | 0.6561 |
| 12 | rs139487710 | T | C | REC | 5/591 | 12/1684 | 0.1034 | 1 | 0.7478 |
| 12 | rs1690916 | A | G | ALLELIC | 211/981 | 776/2616 | 13.99 | 1 | 0.000184 |
| 12 | rs1690916 | A | G | TREND | 211/981 | 776/2616 | 14 | 1 | 0.000183 |
| 12 | rs1690916 | A | G | DOM | 198/398 | 683/1013 | 9.263 | 1 | 0.002339 |
| 12 | rs1690916 | A | G | REC | 13/583 | 93/1603 | 10.9 | 1 | 0.00096 |
| 12 | rs1695143 | G | C | ALLELIC | 244/948 | 708/2684 | 0.08699 | 1 | 0.768 |
| 12 | rs1695143 | G | C | TREND | 244/948 | 708/2684 | 0.08582 | 1 | 0.7696 |
| 12 | rs1695143 | G | C | DOM | 219/377 | 629/1067 | 0.02217 | 1 | 0.8816 |
| 12 | rs1695143 | G | C | REC | 25/571 | 79/1617 | 0.2186 | 1 | 0.6401 |
| 12 | rs2279744 | T | G | DOM | 395/201 | 1177/519 | 1.997 | 1 | 0.1576 |
| 12 | rs2279744 | T | G | REC | 91/505 | 332/1364 | 5.436 | 1 | 0.01972 |
| 12 | rs2279744 | T | G | ALLELIC | 486/706 | 1509/1883 | 4.953 | 1 | 0.02605 |
| 12 | rs2279744 | T | G | TREND | 486/706 | 1509/1883 | 5.053 | 1 | 0.02459 |
| 12 | rs2291857 | T | G | DOM | 339/257 | 958/738 | 0.02778 | 1 | 0.8676 |
| 12 | rs2291857 | T | G | REC | 77/519 | 205/1491 | 0.2831 | 1 | 0.5947 |
| 12 | rs2291857 | T | G | ALLELIC | 416/776 | 1163/2229 | 0.1467 | 1 | 0.7017 |
| 12 | rs2291857 | T | G | TREND | 416/776 | 1163/2229 | 0.1439 | 1 | 0.7045 |
| 12 | rs3730572 | C | G | DOM | 92/504 | 249/1447 | 0.1983 | 1 | 0.6561 |
| 12 | rs3730572 | C | G | REC | 5/591 | 12/1684 | 0.1034 | 1 | 0.7478 |
| 12 | rs3730572 | C | G | ALLELIC | 97/1095 | 261/3131 | 0.2404 | 1 | 0.6239 |
| 12 | rs3730572 | C | G | TREND | 97/1095 | 261/3131 | 0.2361 | 1 | 0.627 |
| 12 | rs3730597 | G | C | DOM | 229/367 | 650/1046 | 0.001768 | 1 | 0.9665 |
| 12 | rs3730597 | G | C | REC | 31/565 | 84/1612 | 0.05715 | 1 | 0.8111 |
| 12 | rs3730597 | G | C | ALLELIC | 260/932 | 734/2658 | 0.01553 | 1 | 0.9008 |
| 12 | rs3730597 | G | C | TREND | 260/932 | 734/2658 | 0.01525 | 1 | 0.9017 |
| 12 | rs4913469 | G | T | DOM | 164/432 | 478/1218 | 0.09736 | 1 | 0.755 |
| 12 | rs4913469 | G | T | REC | 10/586 | 37/1659 | 0.5572 | 1 | 0.4554 |
| 12 | rs4913469 | G | T | ALLELIC | 174/1018 | 515/2877 | 0.2367 | 1 | 0.6266 |
| 12 | rs4913469 | G | T | TREND | 174/1018 | 515/2877 | 0.2407 | 1 | 0.6237 |
| 12 | rs78064292 | A | G | DOM | 141/455 | 399/1297 | 0.004252 | 1 | 0.948 |
| 12 | rs78064292 | A | G | REC | 9/587 | 22/1674 | 0.1498 | 1 | 0.6987 |
| 12 | rs78064292 | A | G | ALLELIC | 150/1042 | 421/2971 | 0.02402 | 1 | 0.8768 |
| 12 | rs78064292 | A | G | TREND | 150/1042 | 421/2971 | 0.02447 | 1 | 0.8757 |
| 20 | rs1100 | A | G | ALLELIC | 360/832 | 1046/2346 | 0.1677 | 1 | 0.6821 |
| 20 | rs1100 | A | G | TREND | 360/832 | 1046/2346 | 0.1656 | 1 | 0.6841 |
| 20 | rs1100 | A | G | DOM | 305/291 | 879/817 | 0.07538 | 1 | 0.7837 |
| 20 | rs1100 | A | G | REC | 55/541 | 167/1529 | 0.1929 | 1 | 0.6605 |
| 20 | rs3761243 | C | A | ALLELIC | 622/570 | 1568/1824 | 12.54 | 1 | 0.000399 |
| 20 | rs3761243 | C | A | TREND | 622/570 | 1568/1824 | 12.07 | 1 | 0.000511 |
| 20 | rs3761243 | C | A | DOM | 454/142 | 1191/505 | 7.708 | 1 | 0.005499 |
| 20 | rs3761243 | C | A | REC | 168/428 | 377/1319 | 8.641 | 1 | 0.003287 |
| 20 | rs60692390 | G | T | DOM | 344/252 | 968/728 | 0.07442 | 1 | 0.785 |
| 20 | rs60692390 | G | T | REC | 70/526 | 191/1505 | 0.102 | 1 | 0.7494 |
| 20 | rs60692390 | G | T | ALLELIC | 414/778 | 1159/2233 | 0.124 | 1 | 0.7247 |
| 20 | rs60692390 | G | T | TREND | 414/778 | 1159/2233 | 0.1262 | 1 | 0.7224 |
| 20 | rs6138989 | A | C | DOM | 343/253 | 970/726 | 0.02296 | 1 | 0.8796 |
| 20 | rs6138989 | A | C | REC | 77/519 | 210/1486 | 0.1163 | 1 | 0.7331 |
| 20 | rs6138989 | A | C | ALLELIC | 420/772 | 1180/2212 | 0.07762 | 1 | 0.7805 |
| 20 | rs6138989 | A | C | TREND | 420/772 | 1180/2212 | 0.07648 | 1 | 0.7821 |
| 20 | rs6138992 | C | T | DOM | 129/467 | 370/1326 | 0.007638 | 1 | 0.9304 |
| 20 | rs6138992 | C | T | REC | 7/589 | 24/1672 | 0.1913 | 1 | 0.6618 |
| 20 | rs6138992 | C | T | ALLELIC | 136/1056 | 394/2998 | 0.03667 | 1 | 0.8481 |
| 20 | rs6138992 | C | T | TREND | 136/1056 | 394/2998 | 0.03661 | 1 | 0.8483 |
| 20 | rs676749 | T | A | DOM | 73/523 | 192/1504 | - | - | - |
| 20 | rs676749 | T | A | REC | 4/592 | 9/1687 | - | - | - |
| 20 | rs676749 | T | A | ALLELIC | 77/1115 | 201/3191 | 0.4415 | 1 | 0.5064 |
| 20 | rs676749 | T | A | TREND | 77/1115 | 201/3191 | 0.4266 | 1 | 0.5137 |

CHR: chromosome; A1: minor allele; A2: major allele; AFF: cases; UNAFF: controls; DF: degree of freedom; DOM: dominant model; REC: recessive model.

Supplemental Table S3.eQTL signals for SNP rs3761243 on gene *GNRH2* and SNP rs1690916 on gene *MDM2*.

| GENE | SNP | P-Values | NES | T-statistic | Tissue |
| --- | --- | --- | --- | --- | --- |
| *GNRH2* | rs3761243 | 0.000066 | 0.2400 | 4.10 | Testis |
| *GNRH2* | rs3761243 | 0.0013 | -0.2300 | -3.30 | Ovary |
| *GNRH2* | rs3761243 | 0.0017 | -0.2800 | -3.20 | Adrenal Gland |
| *GNRH2* | rs3761243 | 0.0033 | -0.2900 | -3.00 | Brain - Caudate (basal ganglia) |
| *GNRH2* | rs3761243 | 0.022 | -0.2100 | -2.30 | Spleen |
| *GNRH2* | rs3761243 | 0.053 | -0.1600 | -1.90 | Brain - Hypothalamus |
| *GNRH2* | rs3761243 | 0.053 | -0.1800 | -2.00 | Brain - Nucleus accumbens (basal ganglia) |
| *GNRH2* | rs3761243 | 0.056 | -0.1200 | -1.90 | Esophagus - Mucosa |
| *GNRH2* | rs3761243 | 0.056 | -0.1200 | -1.90 | Heart - Atrial Appendage |
| *GNRH2* | rs3761243 | 0.057 | -0.1300 | -1.90 | Artery - Aorta |
| *GNRH2* | rs3761243 | 0.095 | -0.1700 | -1.70 | Vagina |
| *GNRH2* | rs3761243 | 0.12 | 0.1200 | 1.60 | Brain - Cerebellar Hemisphere |
| *GNRH2* | rs3761243 | 0.12 | -0.1600 | -1.60 | Brain - Frontal Cortex (BA9) |
| *GNRH2* | rs3761243 | 0.12 | -0.1800 | -1.50 | Brain - Spinal cord (cervical c-1) |
| *GNRH2* | rs3761243 | 0.13 | -0.1100 | -1.50 | Colon - Transverse |
| *GNRH2* | rs3761243 | 0.16 | -0.1300 | -1.40 | Brain - Hippocampus |
| *GNRH2* | rs3761243 | 0.17 | -0.0840 | -1.40 | Breast - Mammary Tissue |
| *GNRH2* | rs3761243 | 0.21 | -0.1100 | -1.30 | Pituitary |
| *GNRH2* | rs3761243 | 0.22 | 0.1300 | 1.20 | Brain - Anterior cingulate cortex (BA24) |
| *GNRH2* | rs3761243 | 0.27 | 0.0660 | 1.10 | Nerve - Tibial |
| *GNRH2* | rs3761243 | 0.32 | -0.1200 | -1.00 | Brain - Substantia nigra |
| *GNRH2* | rs3761243 | 0.38 | 0.0600 | 0.88 | Liver |
| *GNRH2* | rs3761243 | 0.39 | -0.0730 | -0.85 | Artery - Coronary |
| *GNRH2* | rs3761243 | 0.4 | -0.0930 | -0.85 | Uterus |
| *GNRH2* | rs3761243 | 0.41 | 0.0340 | 0.82 | Artery - Tibial |
| *GNRH2* | rs3761243 | 0.43 | -0.0490 | -0.79 | Skin - Not Sun Exposed (Suprapubic) |
| *GNRH2* | rs3761243 | 0.46 | 0.0780 | 0.75 | Brain - Putamen (basal ganglia) |
| *GNRH2* | rs3761243 | 0.48 | -0.0530 | -0.70 | Stomach |
| *GNRH2* | rs3761243 | 0.52 | -0.0730 | -0.65 | Cells - EBV-transformed lymphocytes |
| *GNRH2* | rs3761243 | 0.56 | -0.0380 | -0.58 | Esophagus - Muscularis |
| *GNRH2* | rs3761243 | 0.57 | 0.0360 | 0.57 | Lung |
| *GNRH2* | rs3761243 | 0.58 | 0.0280 | 0.55 | Thyroid |
| *GNRH2* | rs3761243 | 0.59 | 0.0560 | 0.54 | Small Intestine - Terminal Ileum |
| *GNRH2* | rs3761243 | 0.62 | -0.0260 | -0.49 | Adipose - Visceral (Omentum) |
| *GNRH2* | rs3761243 | 0.65 | -0.0530 | -0.46 | Brain - Amygdala |
| *GNRH2* | rs3761243 | 0.66 | -0.0400 | -0.44 | Brain - Cortex |
| *GNRH2* | rs3761243 | 0.8 | 0.0170 | 0.26 | Brain - Cerebellum |
| *GNRH2* | rs3761243 | 0.83 | -0.0180 | -0.22 | Pancreas |
| *GNRH2* | rs3761243 | 0.85 | -0.0230 | -0.18 | Minor Salivary Gland |
| *GNRH2* | rs3761243 | 0.86 | -0.0120 | -0.17 | Heart - Left Ventricle |
| *GNRH2* | rs3761243 | 0.87 | 0.0089 | 0.16 | Skin - Sun Exposed (Lower leg) |
| *GNRH2* | rs3761243 | 0.94 | -0.0062 | -0.07 | Colon - Sigmoid |
| *GNRH2* | rs3761243 | 0.95 | 0.0044 | 0.06 | Prostate |
| *GNRH2* | rs3761243 | 0.97 | 0.0022 | 0.04 | Adipose - Subcutaneous |
| *MDM2* | rs1690916 | 1.40E-07 | -0.3700 | -5.70 | Brain - Spinal cord (cervical c-1) |
| *MDM2* | rs1690916 | 0.000019 | 0.0640 | 4.30 | Whole Blood |
| *MDM2* | rs1690916 | 0.00002 | 0.1300 | 4.30 | Adipose - Visceral (Omentum) |
| *MDM2* | rs1690916 | 0.00065 | 0.0660 | 3.40 | Adipose - Subcutaneous |
| *MDM2* | rs1690916 | 0.0049 | 0.0900 | 2.80 | Breast - Mammary Tissue |
| *MDM2* | rs1690916 | 0.0089 | -0.1900 | -2.70 | Brain - Substantia nigra |
| *MDM2* | rs1690916 | 0.02 | -0.1000 | -2.30 | Brain - Cerebellum |
| *MDM2* | rs1690916 | 0.028 | 0.1000 | 2.20 | Brain - Nucleus accumbens (basal ganglia) |
| *MDM2* | rs1690916 | 0.054 | 0.0530 | 1.90 | Muscle - Skeletal |
| *MDM2* | rs1690916 | 0.081 | 0.1200 | 1.80 | Liver |
| *MDM2* | rs1690916 | 0.094 | -0.1100 | -1.70 | Adrenal Gland |
| *MDM2* | rs1690916 | 0.096 | 0.0900 | 1.70 | Spleen |
| *MDM2* | rs1690916 | 0.11 | 0.0450 | 1.60 | Skin - Not Sun Exposed (Suprapubic) |
| *MDM2* | rs1690916 | 0.12 | 0.0350 | 1.60 | Nerve - Tibial |
| *MDM2* | rs1690916 | 0.15 | -0.1200 | -1.50 | Vagina |
| *MDM2* | rs1690916 | 0.17 | 0.0310 | 1.40 | Artery - Tibial |
| *MDM2* | rs1690916 | 0.21 | 0.0450 | 1.30 | Esophagus - Mucosa |
| *MDM2* | rs1690916 | 0.22 | 0.0950 | 1.20 | Minor Salivary Gland |
| *MDM2* | rs1690916 | 0.22 | -0.0790 | -1.20 | Small Intestine - Terminal Ileum |
| *MDM2* | rs1690916 | 0.22 | 0.0280 | 1.20 | Testis |
| *MDM2* | rs1690916 | 0.26 | 0.0230 | 1.10 | Cells - Cultured fibroblasts |
| *MDM2* | rs1690916 | 0.29 | 0.0440 | 1.10 | Colon - Transverse |
| *MDM2* | rs1690916 | 0.3 | -0.0500 | -1.00 | Pancreas |
| *MDM2* | rs1690916 | 0.31 | 0.0640 | 1.00 | Brain - Anterior cingulate cortex (BA24) |
| *MDM2* | rs1690916 | 0.37 | 0.0450 | 0.90 | Brain - Putamen (basal ganglia) |
| *MDM2* | rs1690916 | 0.38 | 0.0410 | 0.88 | Brain - Frontal Cortex (BA9) |
| *MDM2* | rs1690916 | 0.39 | -0.0870 | -0.86 | Cells - EBV-transformed lymphocytes |
| *MDM2* | rs1690916 | 0.42 | 0.0280 | 0.82 | Esophagus - Muscularis |
| *MDM2* | rs1690916 | 0.45 | -0.0410 | -0.76 | Artery - Coronary |
| *MDM2* | rs1690916 | 0.47 | 0.0320 | 0.72 | Brain - Caudate (basal ganglia) |
| *MDM2* | rs1690916 | 0.51 | -0.0230 | -0.65 | Artery - Aorta |
| *MDM2* | rs1690916 | 0.57 | 0.0360 | 0.57 | Brain - Amygdala |
| *MDM2* | rs1690916 | 0.57 | -0.0300 | -0.57 | Brain - Cerebellar Hemisphere |
| *MDM2* | rs1690916 | 0.58 | 0.0270 | 0.55 | Pituitary |
| *MDM2* | rs1690916 | 0.64 | 0.0160 | 0.46 | Heart - Atrial Appendage |
| *MDM2* | rs1690916 | 0.71 | -0.0190 | -0.37 | Brain - Hypothalamus |
| *MDM2* | rs1690916 | 0.71 | -0.0300 | -0.37 | Ovary |
| *MDM2* | rs1690916 | 0.73 | -0.0160 | -0.34 | Colon - Sigmoid |
| *MDM2* | rs1690916 | 0.77 | -0.0120 | -0.30 | Brain - Hippocampus |
| *MDM2* | rs1690916 | 0.78 | -0.0150 | -0.29 | Brain - Cortex |
| *MDM2* | rs1690916 | 0.79 | 0.0150 | 0.26 | Prostate |
| *MDM2* | rs1690916 | 0.8 | -0.0061 | -0.25 | Skin - Sun Exposed (Lower leg) |
| *MDM2* | rs1690916 | 0.8 | 0.0220 | 0.26 | Uterus |
| *MDM2* | rs1690916 | 0.88 | 0.0064 | 0.15 | Stomach |
| *MDM2* | rs1690916 | 0.92 | -0.0037 | -0.10 | Heart - Left Ventricle |
| *MDM2* | rs1690916 | 0.95 | 0.0018 | 0.06 | Thyroid |
| *MDM2* | rs1690916 | 0.96 | 0.0014 | 0.05 | Lung |

NES: normalized effect size.


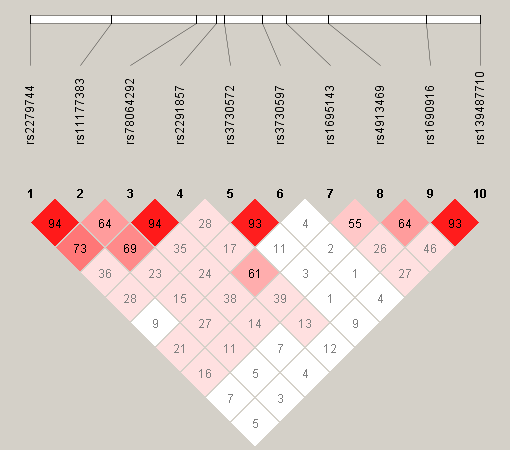


Supplemental Figure S1. Linkage disequilibrium of selected SNPs for *MDM2*. Values of D' were indicated in each square.


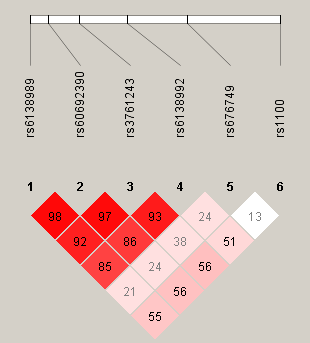


Supplemental Figure S2. Linkage disequilibrium of selected SNPs for *GNRH2*. Values of D' were indicated in each square.
